# Association of Matrix Metalloproteinases with Coronary Artery Calcification in Patients with CHD

**DOI:** 10.3390/jpm11060506

**Published:** 2021-06-03

**Authors:** Yana V. Polonskaya, Elena V. Kashtanova, Ivan S. Murashov, Evgenia V. Striukova, Alexey V. Kurguzov, Ekaterina M. Stakhneva, Viktoria S. Shramko, Nikolay A. Maslatsov, Aleksandr M. Chernyavsky, Yulia I. Ragino

**Affiliations:** 1 Research Institute of Internal and Preventive Medicine—Branch of the Institute of Cytology and Genetics, Siberian Branch of Russian Academy of Sciences (IIPM–Branch of IC&G SB RAS), 175/1 B. Bogatkova Str., 630089 Novosibirsk, Russia; elekastanova@yandex.ru (E.V.K.); stryukova.j@mail.ru (E.V.S.); stahneva@yandex.ru (E.M.S.); nosova@211.ru (V.S.S.); maslatsoff@mail.ru (N.A.M.); ragino@mail.ru (Y.I.R.); 2 The Federal State Budgetary Institution “National Medical Research Center Named Academician E.N. Meshalkin” of the Ministry of Health of the Russian Federation, Rechkunovskaya Str., 15, 630055 Novosibirsk, Russia; ivmurashov@gmail.com (I.S.M.); aleksey_kurguzov@mail.ru (A.V.K.); amchern@mail.ru (A.M.C.)

**Keywords:** metalloproteinases, calcification, atherosclerosis, multiplex assay, coronary heart disease

## Abstract

This work is aimed at studying the relationship of matrix metalloproteinases with calcification of the coronary arteries. The study included 78 people with coronary heart disease (CHD) and 36 without CHD. Blood and samples of coronary arteries obtained as a result of endarterectomy were examined. Serum levels of metalloproteinases (MMP) MMP-1, MMP-2, MMP-3, MMP-7, MMP-9, MMP-10, MMP-12, and MMP-13 were determined by multiplex analysis. In blood vessel samples, MMP-1, MMP-3, MMP-7, and MMP-9 were determined by enzyme immunoassay; MMP-9 expression was evaluated by immunohistochemistry. Patients with CHD had higher serum levels of MMP-1, MMP-7, and MMP-12. Blood levels of MMP-1 and MMP-3 were associated with calcium levels, MMP-9 with osteoprotegerin and osteonectin, MMP-7 and MMP-10 with osteoprotegerin, MMP-12 with osteocalcin, and MMP-13 with osteopontin. Calcified plaques had higher levels of MMP-1 and MMP-9 compared to plaques without calcification. The relative risk of coronary arteries calcification was associated with MMP-9, which is confirmed by the results of immunohistochemistry. The results obtained indicate the participation of some MMPs, and especially MMP-9, in the calcification processes. The study can serve as a basis for the further study of the possibility of using MMP-1, MMP-7 and MMP-12 as potential biomarkers of CHD.

## 1. Introduction

Cardiovascular diseases (CVD) are considered the leading causes of morbidity and mortality worldwide [1]. Calcification of the vascular wall, which contributes to a decrease in vascular elasticity, is one of the leading factors in CVD. The initial stages of calcification are partly related to elastin degradation with the formation of mineral deposits. Metalloproteinases (MMP) play a significant role in the degradation of elastin; MMP-2 and MMP-9 bind to insoluble elastin and break it down to soluble elastin peptides, which bind to receptors on the surface of vascular smooth muscle cells [2], contributing to their osteogenic differentiation. There is an increase in the expression of MMP in smooth muscle cells and macrophages in atherosclerosis, which leads to the destabilization of the atherosclerotic process in the vascular wall. A particularly high level of MMP is observed in the accumulation of foam cells area and the area of the shoulder of the plaque [3,4,5,6]. MMPs are involved in the development and progression of atherosclerosis, and changes in their level are associated with an increased risk of cardiovascular morbidity and mortality [7,8,9].

Studies on the relationship of MMPs with the calcification process are few. Therefore, according to Gaubatz J. W. et al. [10], the positive association of MMP-7 with the calcification of the carotid arteries was revealed. Experimental studies in mice have shown that MMP-2 deficiency contributes to atherosclerotic calcification development [11]. A study on cell cultures showed that gelatinases promote calcification by stimulating osteoblastic differentiation of vascular smooth muscle cells [12]. Extracellular vesicles, secreted by various cells, have been shown to transport and activate MMP and promote vascular microcalcification. The cellular and molecular mechanisms of MMP’s effect on atherosclerotic plaque calcification require further study, which will improve the possible therapy aimed at stabilizing the plaque by reducing the level of its calcification. Therefore, our work aimed to study the relationship of matrix metalloproteinases with coronary arteries calcification.

## 2. Materials and Methods

The study was conducted jointly with the E.N. Meshalkin National Medical Research Center of the Ministry of Health of the Russian Federation and approved by the Ethical Committees of both institutions. The study included 114 people. All patients completed an Informed Consent form.

The core group included 78 men admitted to the National Medical Research Center named after academician E.N. Meshalkin for coronary bypass surgery. The excluding criteria were myocardial infarction less than six months, acute and exacerbation of chronic infections and inflammatory diseases, renal failure, active liver diseases, oncological diseases, hyperparathyroidism. Blood sampling was performed 12 h after the meal, before the operation. During the surgical procedure, according to intraoperative indications, an endarterectomy was performed from the coronary artery (s), after which the surgical material delivered to the histological laboratory within 30 min was divided longitudinally and transversely into fragments for histological and biochemical studies. Macro- and micro-histological analyses of 156 samples of intima/media of the coronary arteries were performed using an Axiostar Plus binocular microscope (C. Zeiss, Munich, Germany) with digital photo output. Fixed tissue samples were dehydrated and embedded in paraffin by means of an automatic embedding device. Serial histological sections (4 µm) were either stained with hematoxylin and eosin (H&E) or were used for immunohistochemistry. Immunohistochemical staining was performed in a LabVision Autostainer 720 (Thermo Scientific, Verona, Fitchburg, MA, USA) according to the UltraVision Quanto HRP DAB protocol with primary polyclonal antibody (Thermo Scientific, Fremont, CA, USA) to MMP-9 (rabbit, polyclonal, ready-to-use) conjugated with horseradish peroxidase. All atherosclerotic plaques samples, depending on calcium deposits presence according to histological analysis results, were divided into (1) samples without calcifications-53, (2) samples with calcifications-103.

As a control group, 36 men were taken from a population sample of Novosibirsk without CHD, comparable to the core group in terms of age and body mass index. Blood sampling was performed 12 h after the meal before the operation.

In the blood we determined the following biochemical calcification factors: (osteoprotegerin (Bender MedSystems, Vienna, Austria), osteocalcin (Immunodiagnostic Systems Ltd., Bensheim, Germany), osteopontin (Bender MedSystems, Vienna, Austria), osteonectin (Immunodiagnostic Systems Ltd., Bensheim, Germany), calcitonin (Biomerica). We did this using the enzyme immunoassay method on a Multiscan analyzer (Finland).

The analysis of MMP concentrations in blood serum was carried out by multiplex analysis on a Luminex MAGPIX flow fluorimeter using two panels (Millipore) manufactured by Merck KGaA (Darmstadt, Germany):

− Panel Milliplex Catalog ID. HMMP1MAG-55K-03, including the determination of matrix metalloproteinase 3 (MMP-3), matrix metalloproteinase 12 (MMP-12) and matrix metalloproteinase 13 (MMP-13);
− Panel Milliplex Catalog ID. HMMP2MAG-55K-05, including the determination of matrix metalloproteinase 1 (MMP-1), matrix metalloproteinase 2 (MMP-2), matrix metalloproteinase 7 (MMP-7), matrix metalloproteinase 9 (MMP-9) and matrix metalloproteinase 10 (MMP-10).

For biochemical analyses, the vessel samples obtained during the surgery were frozen in liquid nitrogen and homogenized in a phosphate–salt buffer solution, with the resulting 1% homogenates divided into aliquots. The protein in the homogenates of the samples was measured using the Lowry method. In the homogenates, we calculated the biochemical parameters relative to the protein.

MMP-9 (RD), MMP-3 (Biosource), MMP-1 (RayBiotech), and MMP-7 (BCM Diagnostics kits) were determined in the homogenates of intima/copper samples of coronary arteries by enzyme immunoassay.

We used the licensed version of the SPSS program (13.0) to perform the statistical processing of the results. The normality of the distribution of biomarkers was determined using the Kolmogorov–Smirnov test. Under normal distribution, the data were presented as M ± SD. Since most biomarkers did not have a normal distribution, we used nonparametric criteria. The results are presented as the 25th, 50th, and 75th percentiles. The significance of the differences was evaluated using the Mann–Whitney test and the chi-square test for categorical variables. We carried out one-factor correlation analysis (Spearman’s method). We used multivariate logistic regression analysis to determine independent predictors of coronary artery calcification. The differences were considered statistically significant at *p* < 0.05.

## 3. Results

Table 1 presents the initial characteristics of patients in the core group.

**Figure 1:**
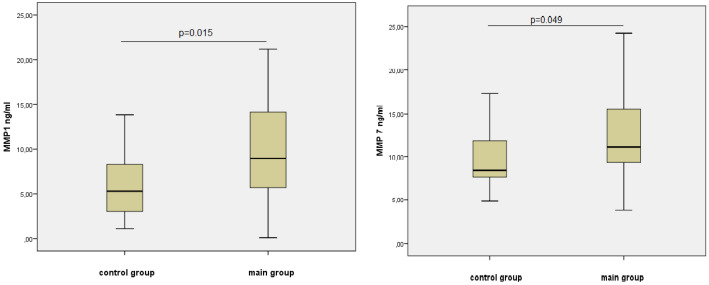
The concentration of MMP-1 and MMP-7 in the studied group, Me (25%; 75%).

**Figure 2:**
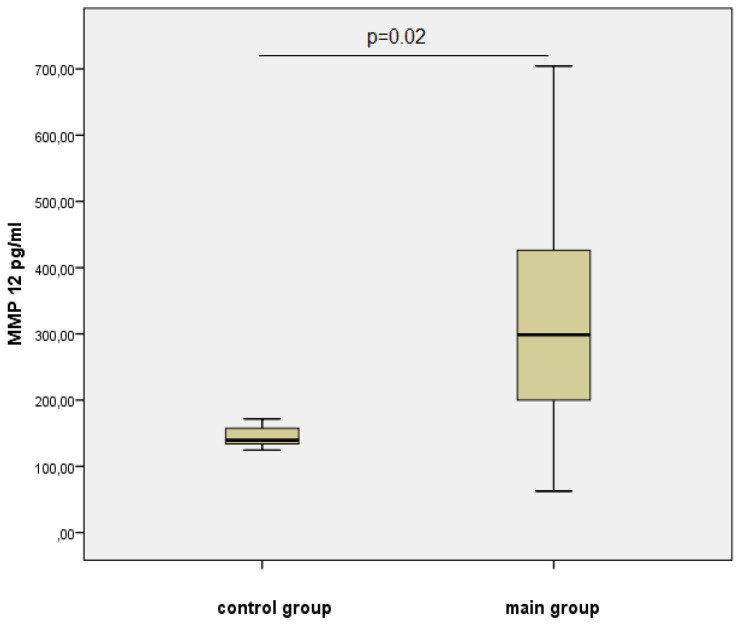
The level of MMP-12 in the studied groups, Me (25%; 75%).

Table 2 and Figure 1 and Figure 2 present the data of the multiplex analysis on the metalloproteinases level for the control group and the group with verified coronary atherosclerosis.

For MMP-1, MMP-7, and MMP-12, we obtained significant differences (Figure 1 and Figure 2). Thus, the level of MMP-1, which is involved in the degradation of collagen in inflammatory diseases and activates MMP-2 and MMP-9, was 1.7 times higher in men of the core group. The content of MMP-7, secreted by epithelial cells and involved in the utilization of extracellular matrix proteins and the activation of pro-MMP-1, -2, and -9, in the serum of men in this group was also 1.3 times higher (Figure 1).

The level of MMP-12, which can hydrolyze elastin and intercellular matrix proteins, as well as activating MMP-3 and MMP-2, was statistically significantly higher in the group of men with atherosclerosis—2.1 times compared to the control group (Figure 2).

At the next stage, we analyzed the relationships of the most significant metalloproteinases with risk factors for cardiovascular diseases. The results are presented in Table 3.

To assess the relationship of metalloproteinases with calcification, we performed correlation analyses with the biochemical factors of calcification and with the presence of calcified plaques in the coronary arteries. There was no association with the presence of plaques, but there was an association of MMP-1 and MMP-3 levels in the blood with calcium levels (r = −0.438; *p* = 0.005 and r = −0.345; *p* = 0.034, respectively); MMP-7 with osteoprotegerin (r = 0.337; *p* = 0.019); MMP-9 with osteoprotegerin (r = −0.414; *p* = 0.001) and with osteonectin (r = 0.409; *p* = 0.011); MMP-10 with osteoprotegerin (r = 0.366; *p* = 0.011); MMP-12 with osteocalcin (r = 0.354; *p* = 0.032); MMP-13 with osteopontin (r = 0.661; *p* = 0.0001).

In the next stage of our study, we examined some of the studied metalloproteinases in atherosclerotic plaques with and without calcifications. Table 4 presents the results.

In calcified plaques, the levels of MMP-9 and MMP-1 were significantly higher by 1.61 and 1.45 times, respectively, compared to plaques without calcification.

When performing a multivariate logistic regression analysis, where the presence/absence of calcification in the atherosclerotic plaque is taken as a dependent variable and the studied MMPs are taken as independent variables, the relative risk of calcification formation in the coronary artery was associated with MMP-9 (Exp (B) = 1.458; 95% CI 1.049–2.027; *p* = 0.025).

**Figure 3:**
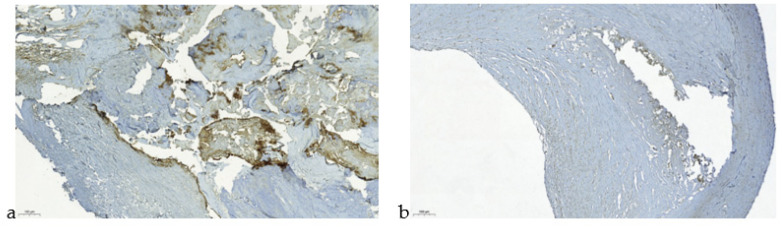
Atherosclerotic plaques of coronary arteries. (**a**) The unstable atherosclerotic plaque with calcification (magnification 100×; MMP9 immunostaining). MMP-9 expression in necrotic areas in the extracellular matrix along the periphery of the calcification core, and in thinned and damaged parts of the fine fibrous cap. (**b**) The stable atherosclerotic plaque without calcification (magnification 100×; MMP9 immunostaining). Lacks MMP-9 expression both in the atheromatous core and in the fibrous cap.

The obtained results have been confirmed by immunohistochemical analysis (Figure 3).

The immunohistochemical investigation of unstable atherosclerotic plaques with calcification showed MMP-9 expression in the necrotic areas in the extracellular matrix along the periphery of the calcification core, and also in thinned and damaged parts of the fine fibrous cap (Figure 3a). The stable atherosclerotic plaque without calcification lacks MMP-9 expression both in the atheromatous core and in the fibrous cap (Figure 3b).

## 4. Discussion

According to the multiplex analysis, the blood levels of MMP-1, MMP-7 and MMP-12 were higher in patients with CHD, which is consistent with the data in the literature. Thus, according to Kondapalli MS et al., elevated levels of MMP-1 in the blood were observed in patients with CHD [13]. The study by Lehrke M. et al., showed that the circulating MMP-1 level may be a possible prognostic marker of the presence of the atherosclerotic plaques, but not a marker of calcification [14]. In the work of Gaubatz J. W. et al., plasma MMP-1 levels were positively associated with carotid artery calcification [10]. In our study, the level of MMP-1 was higher in calcified plaques, although the subsequent logistic regression analysis did not show the effect of MMP-1 on the formation of calcified foci. Since most of the calcified plaques in our study were unstable, this suggests that MMP-1 may play a role in the destabilization of the atherosclerotic plaque. These findings are consistent with those of Cavusoglu E. et al., who found that elevated blood levels of MMP-1 are associated with an increased risk of all-cause mortality in the long-term in patients with CHD. This association was independent of other clinical, angiographic, and laboratory variables [15].

In the literature, there are data on the association of MMP-3 with vascular calcification [16]. According to our data, there was no association of MMP-3 with vascular calcification, but MMP-3 showed an inverse relationship with the level of calcium in the blood. We found no association of MMP-3 with osteopontin, although, according to Fedarko NS et al., osteopontin binds proMMP-3 and active MMP-3 [17].

Goncalves I et al., have demonstrated an association between CVD and increased circulating MMP-7 and -12 levels [18]. According to other authors, patients with carotid artery atherosclerosis had increased plasma levels of MMP-7 compared to healthy people [19]. In our study, MMP-7 and MMP-12 levels were also significantly higher in patients with CHD and verified coronary artery atherosclerosis than in the control group. In a Gaubatz J. W. et al., study, plasma MMP-1 levels were positively associated with carotid artery calcification [10]. The authors found a significant relationship between the MMP-7 level in plasma and the area of carotid artery calcification, which suggests a possible role of MMP-7 in vascular calcification [10]. We obtained data on the relationship of MMP-7 and MMP-12 levels in the blood with calcification factors. Thus, the serum level of MMP-7 showed an association with osteoprotegerin and MMP-12-with osteocalcin.

In terms of serum levels of MMP-9 and MMP-3, we did not find a statistically significant difference between patients with CHD and the control group. Although, according to Ben Braiek A et al., blood levels of MMP-3 and MMP-9 in patients with CHD were significantly higher [20]. In the study by Wu HD et al., and Moradi N et al., elevated serum levels of MMP-9 were also associated with CHD [21,22]. When studying MMP-9 in samples of atherosclerotic plaques, we found that the relative risk of calcification in the coronary artery was associated with MMP-9. This is consistent with the data of Yajie Chen et al., which indicated an association of MMP-9 with vascular calcification [23]. The mechanisms of influence of MMP-9 on calcification can be different. The increased expression of MMP-9 may lead to an osteogenic transformation of smooth muscle cells and macrophages, and may increase the infiltration of monocytes/macrophages into the affected area of the vessel, promoting calcification through secreted microvesicles [23,24,25].

The level of MMP-12, an elastolytic metalloproteinase, in our study was higher in patients with CHD. MMP-12 is activated in atherosclerotic lesions and aneurysms and can promote the activation of other MMPs, which, in turn, destroy other proteins of the extracellular matrix [26].

Thus, the broad and diverse function of these destructive MMPs underscores the importance of expanding our understanding of the role of these destructive proteinases in cardiovascular diseases, which will significantly expand our understanding of the pathogenetic mechanisms of the development of coronary atherosclerosis and, in particular, the calcification of atherosclerotic plaques.

## 5. Conclusions

The results obtained indicate the involvement of some MMPs, especially MMP-9, in the processes of calcification, which requires additional research on the role of MMPs in vascular calcification and atherosclerosis development. The study can serve as a basis for the further study of the possibility of using MMP-1, MMP-7 and MMP-12 as potential CHD biomarkers.

## Figures and Tables

**Table 1:** Characteristics of patients of the core group.

Parameters	Meaning
Clinical and anamnestic characteristics	
Age, years (M ± SD)	60.4 ± 6.3
Body mass index, kg/m2 (M ± SD)	29.3 ± 4.7
Systolic pressure, mmHg (M ± SD)	137.7 ± 12.8
Diastolic pressure, mmHg (M ± SD)	84.9 ± 7.3
Heart rate, beats per minute (M ± SD)	69.7 ± 6.81
The history of heart attack (absolute in %)	69.2
The history of type II diabetes (absolute in %)	11.5
The family history of CHD (absolute in %)	41.3
Smoking (absolute in %)	15.4
Angina pectoris	
Functional Class I	0%
Functional Class II	10.3%
Functional Class III	83.3%
Functional Class IV	6.4%
Multivessel atherosclerotic lesion	
of coronary arteries (more than two vessels)	92.3%
Biochemical parameters, Me (25%; 75%)	
Calcitonin, (pg/mL)	1.86 (0.02; 2.98)
Osteoprotegerin, (pg/mL)	52.99 (35.43; 79.95)
Osteopontin, (ng/mL)	27.05 (17.62; 39.61)
Osteocalcin, (ng/mL)	13.26 (8.46; 16.63)
Osteonectin, (µg/mL)	8.96 (7.74; 10.72)
Ca (mol/L)	2.3 (2.22; 2.43)

**Table 2:** The level of destruction markers in the blood of patients. Me (25%; 75%).

Parameters	Control *n* = 36	Group with CHD *n* = 78	*p*
MMP-2 (ng/mL)	111.68 (89.14; 126.89)	104.28 (78.71; 120.04)	0.422
MMP-3 (ng/mL)	47.1 (28.4; 66.5)	33.44 (21.11; 63.65)	0.311
MMP-9 (ng/mL)	228.0 (130.18; 354.96)	276.01 (151.31; 327.58)	0.342
MMP-10 (ng/mL)	0.62 (0.52; 0.82)	0.68 (0.51; 0.82)	0.785
MMP-13 (pg/mL)	30.35 (17.56; 71.9)	36.37 (17.28; 58.16)	0.739

**Table 3:** The level of metalloproteinases, depending on the risk factors for cardiovascular diseases in men with CHD.

Parameters		MMP-1 (ng/mL)		MMP-7 (ng/mL)		MMP-12 (pg/mL)	
			*p*		*p*		*p*
BMI	<25	7.96 (4.61; 15.78)	0.97	9.36 (8.25; 12.31)	0.65	218.7 (116.9; 338.5)	0.10
	>25	7.74 (5.49; 11.84)		11.07 (9.36; 15.86)		233.6 (149.6; 382.8)	
Smoking	no	9.22 (6.18; 18.37)	0.52	11.48 (9.36;15.09)	0.08	299.5 (208.6; 513.6)	0.67
	yes	8.37 (2.11; 20.29)		15.09 (8.92; 17.36)		200.8 (101.2; 354.5)	
Family history of CHD	no	10.71 (7.64; 22.78)	0.04	12.72 (9.36; 16.05)	0.53	298.7 (205.3; 554.9)	0.19
	yes	7.41 (5.36; 13.26)		10.22 (9.36; 15.09)		301.4 (174.0; 393.2)	

**Table 4:** The level of metalloproteinases in atherosclerotic foci.

Parameters	With Calcification *n* = 103	Without Calcification *n* = 53	*p*
MMP-9 (ng/mg of protein)	3.61 (1.62; 5.08)	2.24 (1.22; 4.16)	0.017
MMP-3 (ng/mg of protein)	2.05 (1.52; 3.3)	2.00 (1.24; 4.38)	0.881
MMP-7 (ng/mg of protein)	0.78 (0.27; 2.41)	0.62 (0.29; 1.56)	0.6
MMP-1 (ng/mg of protein)	71.37 (21.09;154.99)	49.21 (6.05;299.3)	0.048

## Data Availability

The datasets before and after analysis in this study are available from the corresponding author on reasonable request.
